# Prevalence of amyloid deposition in long standing rheumatoid arthritis in Iranian patients by abdominal subcutaneous fat biopsy and assessment of clinical and laboratory characteristics

**DOI:** 10.1186/1471-2474-7-43

**Published:** 2006-05-15

**Authors:** G Hussein Alishiri, Ahmad Salimzadeh, Mohammad Bagher Owlia, Jafar Forghanizadeh, Roya Setarehshenas, Nasrin Shayanfar

**Affiliations:** 1 Assistant Professor of Medicine, Department of Rheumatology, Baqyatollah University of Medical Sciences, Tehran, Iran; 2 Assistant Professor of Medicine, Department of Rheumatology, Tehran University of Medical Sciences, Tehran, Iran; 3 Assistant Professor of Medicine, Department of Rheumatology, Shahid Sadoughi University of Medical Sciences, Yazd, Iran; 4 Professor of Medicine, Department of Rheumatology, Iran University of Medical Sciences, Tehran, Iran; 5 Assistant Professor of Medicine, Department of Pathology, Iran University of Medical Sciences, Tehran, Iran

## Abstract

**Background:**

The study was aimed at determining the prevalence of secondary amyloidosis in a group of Iranian patients with Rheumatoid Arthritis (RA), and the assessment of its correlation with the clinical and laboratory findings and data.

**Method:**

A total number of 220 patients (167 female and 53 male) with a minimum five-year history of RA were selected. Congo red staining method was used for staining the specimens obtained by abdominal subcutaneous fat biopsy (ASFB) method. All of the specimens were examined for apple-green birefringence under polarized light microscope. Clinical and laboratory characteristics of the patients were assessed. Chi-square test and unpaired student's t-test were run for intergroup comparisons.

**Results:**

Amyloid deposition test yielded positive results in 15 out of the 220 cases (6.8%) examined by the ASFB technique. Thirteen patients were found to have minimal amyloid deposits. Of all the clinically significant cases, 8 (53%) presented with proteinuria, and 7 cases (46.6%) had severe constipation.

**Conclusion:**

The prevalence of fat amyloid deposits in Iranian patients with RA is low. In up to half of the study group the deposits were subclinical. Follow up studies are required to determine whether this subclinical amyloidosis can develop into full-blown clinically significant amyloidosis.

## Background

Reactive or secondary amyloidosis is a well-known significant late complication of chronic inflammatory disease, especially rheumatoid arthritis (RA) [1].

In this condition, large amounts of amyloid molecules (of the AA type) are deposited over a wide area. The main clinical manifestations of secondary amyloidosis (SA) are marked proteinuria and gastrointestinal symptoms. Generally, the disease carries a poor prognosis, and causes death in 2–9% of the cases [2,3].

The prevalence of SA in RA patients in western countries varies between 8 and 54%, according to the ethnicity of the subject, the method of amyloid detection, and other variables in the patients' condition [4].

The simplest most reliable method for amyloid deposition screening, which is considered gold standard procedure, is abdominal subcutaneous fat aspiration (ASFA) method. Congo red staining is used in the procedure [5]. The abdominal fat pad aspiration method has a sensitivity of 26.5 and 82% for the detection of amyloid deposits in RA cases and in sure amyloidosis cases respectively [6-9].

Another method, that is other than the ASFA method, namely the biopsy technique, has been given clinical trial to develop a more sensitive method for detecting amyloid deposits [10-12]. Barile et al showed that tru cut needle biopsy has a sensitivity of 78% for amyloid detection. The study did not show any correlation between amyloid deposition and the clinical manifestations of the disease [10]. Breedveld obtained 54% positive results using the biopsy method rather than the aspiration method in a diverse inflammatory arthritic group, and with samples taken from different sites[11].

In the two studies mentioned, the selected subjects were too diverse in their clinical picture and/or too few for any definitive conclusion to be drawn from the test results that were obtained. It would therefore be quite reasonable to conclude that the biopsy method affords greater sensitivity to amyloid deposition; therefore, it yields more accurate results compared with the aspiration method in the detection of amyloid deposits.

A variety of studies have been conducted to determine the prevalence of secondary amyloidosis in RA patients; they have yielded varying results in accordance with the type of geographic population studied and the diagnostic method used [12]. The data, published as case series or case reports, show that the prevalence of secondary amyloidosis in the Middle Eastern region, and in the neighboring countries of Iran, is low [13,14]. Our study, which to the best of our knowledge is the first of its kind, was directly aimed at investigating the prevalence of amyloid deposition in Iranian patients using fat-pad biopsy method; it also sought to establish whether and how its occurrence correlated with the clinical and laboratory findings and data respectively.

## Methods

### Participants

The subjects were selected from a pool of patients referred to two out-patient clinics of Rasool Akram and Baqiyatollah hospitals in Tehran, Iran, within the period of December 2001 through October 2003.

### Inclusions

All of the subjects fulfilled the 1987 American College of Rheumatology (ACR) classification criteria for RA [15]. They were included in the study consecutively. The duration of the disease for all the subjects was longer than 5 years, and their disease was still active. All the patients had been referred to the aforementioned clinics by specialists.

### Exclusions

Patients with an onset of disease earlier than 16 years of age, coexisting chronic disease itself capable of inducing amyloidosis (e.g. chronic infection), and those otherwise declining to take part in the trial were excluded from the study.

All of the 220 patients that took part in the study gave their written informed consent prior to entering the study. The approval of the Ethics Committee of the Research Department of the University was sought and obtained.

### Method

Small samples (minimum size 3*3 mm) of abdominal fat pad were obtained through a small periumbilical incision, under local induced anesthesia by 1 ml lidocaine 1%. The specimens were fixed in 10% formalin. On the same occasion, an 18 gauge needle connected to a 10 ml syringe was inserted into the periumblical fat tissue. A suction force was applied to aspirate fat samples, which were subsequently mounted on 3 microscope slides, and left to air-dry prior to being fixed in 10% formalin solution as fixative. The entire procedure on all the subjects was performed by the same doctor. The samples were subsequently stained with Congo red stain and examined under polarized light microscope. Two independent pathologists blindly examined the specimens.

The intensity of the staining was assessed by visual estimation in all the 3 samples for each patient. The amounts of amyloid deposits found were rated as: negative for no detectable amyloid deposits in a small isolated area, mild (+) for little, less than 10 % involvement, moderate (++) for amyloid deposition between 10 and 60 % involvement, and severe (+++) for involvement of over 60% of the test area. Where any conflict occurred between the examiners' test results, the smallest value was considered.

Patient variables, including age, disease duration, age at the onset of the disease, presence or absence of constipation, and the use of disease-modifying anti-rheumatic drugs (DMARDs), such as gold and D-penicillamine, were noted and duly taken into account. Muscle strength was measured by 5 degree force score normally applied during routine physical examination [16]. Functional disability status was evaluated by revised criteria for the classification of global functional status [17]. Radiographic damage was evaluated according to the Steinbrocker criteria for the classification of the progression of rheumatoid arthritis. Thorough physical examination was conducted and complete blood count, ESR, IgM rheumatoid factor, and C reactive protein values were obtained and duly taken into account. Urinalysis results and 24-hour urine protein values, where indicated, were also obtained. Plain hand X-rays were obtained for all the patients.

Statistical analysis was performed with spss version ll.5. The chi-squared test and unpaired student t-test were run for intergroup comparisons. A p value of less than 0.05 was considered significant. RA patients with proteinuria and positive results for amyloid deposition, using the ASFB method, were diagnosed with clinical amyloidosis.

## Results

Fat tissue specimens were obtained from all the 220 patients. Amyloid deposition test result was found positive in 15 of the 220 cases. Thirteen of the specimens showed minimal (+) amyloid deposition, with 2 specimens showing moderate (++) amyloid deposition, but none with severe (+++) amyloid deposition.

Ten out of the 15 cases that tested positive for amyloid deposition by biopsy method also tested positive for it by fat aspiration method. Nine out of the 15 (60%) cases had clinical amyloidosis, and presented with marked proteinuria of between 300 mg to 800 mg in 24-hour urine collection (P < 0.05). None of the patients had developed pedal edema, and none was taking gold or D-penicillamine therapy. Proteinuria was also present in 17 out of 205 patients (8%) who tested negative for amyloidosis with the ASFB method. In these patients the intensity of proteinuria was less than 500 mg/24 h. After one year's follow-up study none of the asymptomatic cases were found to have developed any renal abnormalities. Constipation was present more frequently in the patients who tested positive for amyloid deposition (P < 0.05).

No significant difference was found related to such variables as age, sex, or duration of the disease between the patients who tested positive for amyloid deposition and those who tested negative (Table 1).

A greater number of the patients who tested positive for amyloid deposition presented with higher functional disability class than the ones that tested negative for amyloid deposition (P < 0.05). No significant difference in laboratory findings was found between the patients that tested positive for amyloid deposition and the ones that tested negative for it, as shown in Table 1.

## Discussion

Secondary amyloidosis is a well-known complication of RA. The prevalence of secondary amyloidosis in rheumatoid arthritis patients varies considerably according to the geographical population studied and the diagnostic method used.

The prevalence of amyloidosis in Western countries varies greatly from region to region [7,10,18]. Recent studies using ASFA method in detecting amyloidosis in Asian and North African countries have also yielded varying results. Wakhlu et al showed in a study that 30 out of 113 (26.5%) adult Asian RA patients from northern India tested positive for amyloid deposition by the ASFA method [9]. Mansouri et al detected amyloid deposits in 8 out of 112 (7%) of the smears taken from Egyptian RA patients using the ASFA method [8] (Table 2).

The prevalence of secondary amyloidosis in the Middle Eastern region and the neighboring countries of Iran has not been established with certainty.

Published data are available as case series or case reports. Ozdogan et al published the case reports of 147 Turkish patients with juvenile chronic arthritis compiled retrospectively [13]. A 10% incidence of secondary amyloidosis was found in the study group. Only a single case of juvenile chronic arthritis and amyloid deposition was reported in a Saudi patient [14].

It seems that the biopsy method yields more positive results than those obtained using the aspiration method. Barile performed an ASFB with a tru-cut needle in 50 Mexican RA patients in order to investigate the presence of secondary amyloidosis in them. Amyloid deposits were found in 78% of the cases, but no correlation was established between amyloid deposition and the clinical manifestations of disease [10]. The largest series of studies was that conducted by Kobayashi et al on a group of Japanese patients [12]. Of the 407 cases studied, 54 (13.3%) were found to have gastrointestinal amyloidosis on gastroduodenal endoscopy.

In our study, a group of consecutive RA patients were examined for evaluation using the SAFB method. A prevalence of 6.8% in amyloid deposition was found in the 220 cases studied with a mean disease duration of 9 years. It is a lower incidence than that obtained in the Mexican and Japanese series using the biopsy method. This disparity can be attributed to a lower prevalence of secondary amyloidosis in the specific geographic population studied.

In a Spanish survey of 313 RA patients with a history of the disease longer than five years, amyloid deposition was detected in 16% of the cases, using the ASFA test [7]. Common clinical features of nephropathy were present in 25% of these amyloid-positive cases. In asymptomatic amyloid-positive patients, long-term follow-up did not show any overt amyloidosis in the majority of the cases. This was found to be the case not only at the time of performing the ASFA test, but also after a long follow-up period.

In our study, 60% of amyloid-positive cases presented with clinical proteinuria. None of the 220 patients studied presented with nephrotic or malabsorption syndromes. Although a statistical difference was found in the severity of the proteinuria (up to 800 mg/24 h) between amyloid-positive and amyloid-negative groups as shown in Table 2, its clinical significance cannot be confirmed at this stage.

A correlation was suggested between the presence of clinical amyloidosis and the intensity of amyloid deposition. Kobayashi et al found that the clinical manifestations suggestive of systemic amyloidosis in gastroduodenal mucosa were more frequent in the group with marked amyloid deposition than in the group that presented with mild amyloid deposition. (47% vs 14%, P < 0.05) [12]. Gómez-Casanovas et al also found the frequency of marked deposition to be significantly higher in the group of patients who presented with visceral amyloidosis (57% vs. 22%) [7].

In our study, 13 of the positive specimens showed minimal and two of them showed moderate amyloid deposition, but none displayed severe amyloid deposition. Eight out of 15 patients had clinical amyloidosis, and presented with proteinuria. No correlation was established between the intensity of amyloid deposition and the clinical manifestations of the disease. The subjects that tested positive have been far too few for any definitive conclusions to be made with regard to the clinical significance of the results.

Significant correlation was suggested between the intensity of the amyloid deposition and the duration of the disease [3]. In the present study, no significant difference was found, as related to duration of disease, between the amyloid-positive and the amyloid-negative groups.

Although the number of the cases studied is too small to draw firm conclusions from the results that were obtained, it suggests constipation and proteinuria to be prominent features of the study group, which consisted entirely of Iranian rheumatoid arthritic patients.

A greater number of patients who tested positive for amyloid deposition presented with more functional disability class and progression stage than the ones who tested negative for amyloid (P < 0.05). No significant difference was found in laboratory findings between the amyloid-positive and the amyloid-negative groups, as shown in Table 1.

## Conclusion

The study showed that a group of Iranian patients with RA presented with low prevalence of amyloid deposition (6.8%); about half of the patients had presented with subclinical disease. Based on the present study and the series mentioned, it seems that a positive amyloid test result per se cannot have much clinical significance, especially as Gómez-Casanovas noted [7,19]. Moreover, a positive test result for amyloid should not prompt the physician to adopt a more invasive treatment choice in order to curtail the progression of the disease. Thus, in our opinion, it does not stand up to logic that a test with such a low prognostic value as that of the ASFB method be adopted or recommended as a screen test.

## Competing interests

The author(s) declare that they have no competing interests.

## Authors' contributions

GHA participated in the design of the study, coordination and carried out the clinical study and performed the statistical analysis

AS participated in the design of the study, helped to accomplish the study, drafted the manuscript, performed final correction and as corresponding author

MBA participated in the design of the study

JF participated in the design of the study

RS helped to pathologic investigation

NS helped to pathologic investigation

All authors read and approved the final manuscript.

## Pre-publication history

The pre-publication history for this paper can be accessed here:



## Figures and Tables

**Table 1:** Comparison of clinical and laboratory variables of amyloid positive and negative patients with rhumatoid arthritis.

		**Amyloid positive**	**Amyloid Negative**	P* value
- Total patient, no (%)		15(6.8)	205(93.2)	-
- Age, yrs, mean ± SD		55.2 ± 12(32–75)	51.6 ± 13.2 (22–80)	NS
- Male: Female, no		3:12	50: 155	NS
- Age at disease onset yrs. Mean ± SD		46.07 ± 7.33 (27–65)	40.18 ± 6.39 (17–70)	NS
- Disease duration, yrs, Mean ± SD		9.13 ± 4.67 (5–20)	11.41 ± 6.85 (5–39)	NS
- Constipation no (%)		7(46.6)	39(19)	<0.019
- Proteinuria, no. (%)		9(60)	17(8.3)	<0.001
- RF. Positive, no. (%)		10(66)	130(63.7)	NS
- CRP, *No, (%)		13(86)	156(67)	NS
- Receiving DMARD, no, (%)		12(80)	155(75.6)	NS
- Past use of gold, no (%)		0	17(8.3)	NS
Functional class	Class 3 No (%)	7	49	< 0.5
	Class 4 No (%)	2	4	

RF: Rheumatoid Factor; CRP: C-reactive protein; DMARD: Disease Modifying Antirheumatic Drug.

*Normal value <2.3 mg/l.

**Table 2:** Comparison of clinical and laboratory variables of amayloidosis.

	Monsoury et al^8 ^(with ASFA)	Wakhlu et al ^9 ^(with ASFA)	Gomez-Casanovas et al^7 ^(with SFA)	Alishiri et al (with ASFB)
No. of patients	112	113	313	220
Positive for amyloid no (%)	8(7)	30(26.5)	51(16%)	15(6.8)
Subclinical amyloid (%)	62.5	73.3	67	46.6
Fat deposit analysis no	8	30	51	15
1+	7	23	12	13
2+	1	5	19	2
3+	0	2	20	0
Duration of disease (year)	>5	>5	>5	>5
Age, yrs, mean ± SD	54(41–65)	46 ± 8.7	61.4 ± 13.7	55.2 ± 12
Male: female	0:8	8:22	1:19	1:3
Disease duration, years mean ± SD	17(12–25)	10 ± (5–22)	16.8 ± 10.5	9.13 ± 4.67(5–20)
Age at disease onset, years ± SD	32.5 (22–50)	36 ± 10	44.9 ± 14.4	46.07 (27–65)
Follow up. Mo, mean ± SD	-	48 ± 36(12–14)	69.7 ± 53.3	12
RF positive, no. (%)	-	25(83.3)	45(75)	10(66.6)
Proteinuria, no (%)	2(25)	7(23.3)	8(13)	8(53.3)

ASFA: Abdominal Subcutaneous Fat Aspiration;

ASFB: Abdominal Subcutaneous Fat Biopsy;

RF: Rheumatoid Factor.
